# Research on Convolutional Neural Network Inference Acceleration and Performance Optimization for Edge Intelligence

**DOI:** 10.3390/s24010240

**Published:** 2023-12-31

**Authors:** Yong Liang, Junwen Tan, Zhisong Xie, Zetao Chen, Daoqian Lin, Zhenhao Yang

**Affiliations:** 1 Key Laboratory of Advanced Manufacturing and Automation Technology (Guilin University of Technology), Education Department of Guangxi Zhuang, Autonomous Region, Guilin 541006, China; void414@163.com; 2 College of Mechanical and Control Engineering, Guilin University of Technology, Guilin 541006, China; xiezs1972@163.com (Z.X.); chenzetao2021@163.com (Z.C.); lindq1997@163.com (D.L.); 19950909667@163.com (Z.Y.)

**Keywords:** FPGA, HLS, edge intelligence, deep learning, heterogeneous computing

## Abstract

In recent years, edge intelligence (EI) has emerged, combining edge computing with AI, and specifically deep learning, to run AI algorithms directly on edge devices. In practical applications, EI faces challenges related to computational power, power consumption, size, and cost, with the primary challenge being the trade-off between computational power and power consumption. This has rendered traditional computing platforms unsustainable, making heterogeneous parallel computing platforms a crucial pathway for implementing EI. In our research, we leveraged the Xilinx Zynq 7000 heterogeneous computing platform, employed high-level synthesis (HLS) for design, and implemented two different accelerators for LeNet-5 using loop unrolling and pipelining optimization techniques. The experimental results show that when running at a clock speed of 100 MHz, the PIPELINE accelerator, compared to the UNROLL accelerator, experiences an 8.09% increase in power consumption but speeds up by 14.972 times, making the PIPELINE accelerator superior in performance. Compared to the CPU, the PIPELINE accelerator reduces power consumption by 91.37% and speeds up by 70.387 times, while compared to the GPU, it reduces power consumption by 93.35%. This study provides two different optimization schemes for edge intelligence applications through design and experimentation and demonstrates the impact of different quantization methods on FPGA resource consumption. These experimental results can provide a reference for practical applications, thereby providing a reference hardware acceleration scheme for edge intelligence applications.

## 1. Introduction

In 2017, AlphaGo’s consecutive victories against human players brought AI into the public eye, sparking a wave of interest. Today, artificial intelligence has been widely applied in various fields, such as in the medical domain for tasks like image denoising [1], ultrasound image processing [2], and image classification [3]. Additionally, significant achievements have been made in areas like small object detection [4] and speech recognition [5]. Currently, most AI computational tasks rely on deployment on cloud and other large-scale computing platforms, but the significant physical distance between these resource-intensive platforms and smart endpoints limits the convenience of AI. This has given rise to the idea of integrating edge computing (EC) with AI, leading to the emergence of edge intelligence (EI) [6,7]. EI enables the transfer of AI technologies from central clouds to the edge, closer to data sources, facilitating low-latency, distributed, and highly reliable services [8].

Convolutional neural networks (CNNs) [9], as one of the typical deep learning algorithms, have achieved significant success compared to other AI algorithms such as support vector machines (SVMs) and decision trees [10] in various computer vision fields, including image classification, semantic segmentation, and object detection [11]. They have been widely applied in various domains, including smart cities [12] and the industrial Internet of Things (IoT) [13]. Due to the large number of parameters and computational complexity of CNNs, research in edge intelligence (EI) has led to the emergence of a series of network lightweighting techniques such as network pruning [14], model distillation [15], parameter quantization [16], and Once-for-All [17]. These techniques aim to reduce a model’s memory and computational requirements, making it suitable for use on low-power and resource-constrained edge devices. In addition to network lightweighting, there is a widespread focus on achieving a balance between computational power and power consumption through hardware acceleration techniques. Traditional computing platforms primarily include CPUs, GPUs, FPGAs, and ASICs. Among these, GPU [18], FPGA [19,20], and ASIC [21] excel in parallel implementations and can be applied to edge-side inference. To better adapt to deep learning intelligent algorithms while considering power constraints on edge devices, the core processor chips of computing platforms often adopt heterogeneous forms. Many single-chip solutions also use customized heterogeneous approaches to further improve energy efficiency [22], mitigating the trade-off between computational power and power consumption. In this context, reference [23] proposed a universal ICU SOC that utilizes a RISC-V-based processor as the command and control platform, with an FPGA as a hardware accelerator, effectively adapting to changing workloads over time. Reference [24] introduced a highly parameterized CGRA accelerator that excels in processing data flow graphs and may be more efficient for specific tasks compared to FPGA.

This article is based on the Zynq device (hardware environment), Vivado HLS 2018.3, and Vivado 2018.3 (software environment). IP cores were generated using synthesis tools in HLS and then deployed on FPGA boards. Compared to Xilinx’s embedded AI development tool, Vitis-AI, this approach is more versatile and flexible, meeting the requirements of non-standard network structure designs. In edge computing devices, in addition to pursuing high performance, reducing power consumption is of vital significance for prolonging device life and reducing heat generation. Different optimization commands in HLS can achieve diverse optimization effects, making it suitable for a wide range of hardware acceleration scenarios. In this article, we tested and compared two different accelerators designed specifically for the high computational complexity of CNN convolution calculations on FPGA platforms, namely the low-performance, low-power UNROLL accelerator and the high-performance, high-power PIPELINE accelerator. We compared the two accelerators in terms of power consumption and resource performance, and analyzed the reasons. Additionally, we compared the throughput and power consumption performance of a CNN running on CPU and GPU platforms with the performance of a CNN running on the FPGA platform in our experiments. The contributions of this article are the following:

- We designed energy-efficient accelerators for the LeNet-5 network using Vivado high-level synthesis (HLS), implementing convolutional calculations, activation, pooling, and fully connected operations on the PL side.
- We applied Gaussian filtering and histogram equalization algorithms on the PS side to perform noise filtering on images, enhancing the differentiation between target characters and background noise, highlighting character details for improved recognition by the Lenet-5 convolutional neural network on the FPGA platform.
- We quantized weight parameters and analyzed resource consumption for different data types to determine the optimal solution. We then transformed our fixed-point quantization into a parameterized quantization to ensure compatibility with various FPGA platforms.
- We designed two different optimization schemes for the convolution calculations and compared our experimental results, demonstrating that the designed accelerators achieved faster speeds and lower power consumption compared to platforms like CPU.

The remaining sections of this paper are organized as follows. Section 2 provides an introduction to relevant work related to this research. Section 3 offers a detailed exposition of image processing algorithms, model optimization strategies, and CNN hardware acceleration approaches. Section 4 describes the system architecture and hardware implementation of the accelerator. Section 5 explores the analysis of experimental results with different accelerators and compares them with other platforms. Section 6 provides a comprehensive summary of the entire work, emphasizing the design’s limitations and future optimization directions.

## 2. Related Work

Since Apple proposed OpenCL for heterogeneous computing in 2008, research on using FPGA-based hardware platforms and ARM+FPGA heterogeneous approaches to accelerate CNNs has become increasingly rich [25]. However, many challenges still exist. CNN computations primarily focus on convolutional layer operations, and multiply–accumulate (MAC) operations are the basic building blocks of fully connected layers and convolutional layers. Therefore, a significant amount of research is concentrated on how to implement new lightweight convolutional operation architectures that ensure computational precision while improving the efficiency of convolutional operations. Current research directions mainly revolve around the following aspects.

Acceleration is achieved through optimizing network structures and quantizing and compressing model parameters. Li et al. [26] introduced an inference engine that can determine the optimal weight quantization bit width of the LeNet network. This engine allows most of the data in the network’s computation process to be quantized using 8 bits and employs 16-bit computation for a small portion of the data, resulting in significant hardware resource savings. Wei et al. [27] explored different approaches for storing network parameters. To minimize external RAM access time, on-chip BRAM was chosen for storing network parameters, and the entire network’s parameters, except for the input and output layers, were binarized. The result achieved a throughput of 0.4 FPS and a power consumption of 2.8 W. Huang et al. [28] proposed deep deformable convolution on the CenterNet network architecture to reduce resource consumption and used ShuffleNet V2 [29] as the network backbone to conduct experiments on the Pascal VOC dataset, achieving a throughput of 26 FPS and a power consumption of 5.6 W.

To accelerate convolutional layer calculations, Zhang [30] and Guan [31] used traditional sliding window convolution algorithms to design deep learning accelerators based on FPGAs. Ahmad and Pasha [32] designed pipeline and parallel convolution computation engines to improve the throughput of convolution calculations while reducing the overall system’s computational complexity and power consumption. Accelerators based on fast algorithms like Winograd and FFT [33,34] achieve rapid convolution computations at the algorithmic level. In [35], a comparison between Winograd and FFT algorithms was made, highlighting that FFT algorithms require more memory due to complex numbers. Under similar parameter configurations, Winograd exhibited better acceleration compared to FFT algorithms. 

Furthermore, Chen Zhang and his colleagues [36] implemented a deep pipelined multi-FPGA architecture to overcome the resource constraints of a single FPGA board. They used a cluster of FPGAs to extend the design space efficiently for accelerating convolutional neural networks. They connected seven FPGA boards using high-speed serial links and proposed a dynamic programming algorithm to deploy the layers of convolutional neural networks effectively across different FPGA boards in the cluster. They successfully implemented AlexNet and VGG-16 neural networks in the FPGA cluster, and the results showed that their approach improved energy efficiency by 21 times and 2 times compared to optimized multi-core CPUs and GPUs, respectively.

## 3. Methodology

### 3.1. Optimization of the LeNet-5 Model

**Figure 1:**
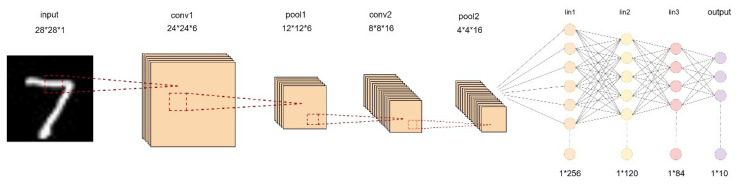
LeNet-5 network architecture.

The structure of the LeNet-5 neural network is shown in Figure 1. The input feature map has a size of 28 × 28 × 1, totaling 784 pixels.

In this study, the classic LeNet-5 model was optimized by introducing dropout into the fully connected layer calculation. Compared with the original algorithm, the optimized algorithm reduces the complexity of the network and is suitable for implementation on FPGA platforms.

**Figure 2:**
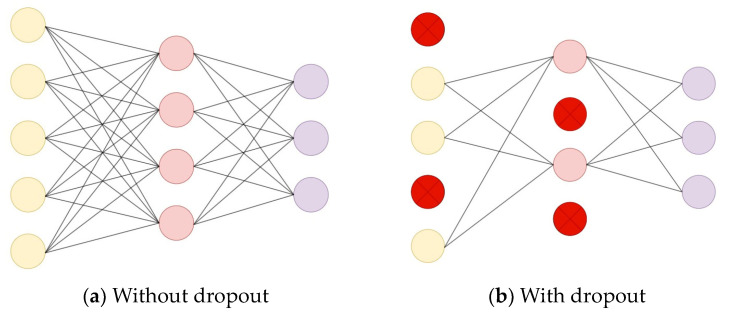
Comparative diagram of the FC network structure before and after adding dropout.

A schematic diagram comparing the network structure before and after adding dropout in the fully connected layer during forward propagation is shown in Figure 2. Dropout randomly deactivates a portion of neurons with a certain probability P during the forward propagation phase. The purpose of dropout is to prevent network overfitting, enhance the network’s generalization performance, and improve its recognition accuracy.

Furthermore, using a compression function can lead to the phenomenon of gradient vanishing in images, resulting in the loss of image features. Therefore, in this study, an activation function was used. The expression is shown in Equation (1).

(1) $$relu\left(x\right)=\left\{\begin{array}{c} x\\ 0 \end{array}\right.\;\begin{array}{c} if\\ if \end{array}\;\begin{array}{c} x>0\\ x\leq 0 \end{array}$$

As shown in Table 1, the LeNet-5 neural network has an input data size of 784 (28 × 28 × 1) without padding. The conv1 has 6 × 1 × 5 × 5 = 150 weights, the conv2 has 16 × 6 × 5 × 5 = 2400 weights, the FC1 has 256 × 120 = 30,720 weights, the FC2 has 120 × 84 = 10,080 weights, and the FC3 has 84 × 10 = 840 weights. Therefore, the total number of weights in the LeNet-5 network is 150 + 2400 + 10,080 + 840 + 2550 = 44,190. The calculated result indicated that 250 KB of SRAM was sufficient to store all weight data of this model, making it suitable for deployment on ZedBoard.

### 3.2. Convolution Calculation

During the operation of the convolutional computation module, the first step was to load the input weight data. Next, the input image data were loaded, and the data entered the row buffer for waiting for convolution computation. Then, the convolution calculation was performed in a sliding window manner, where multiplication and accumulation operations were performed as the window slid. After the computation was complete, the output channels were integrated. The convolution calculation formula is shown as Equation (2).

(2) $$G\left(x,y\right)=\frac{1}{2\pi \sigma^{2}}e^{-\frac{x^{2}+y^{2}}{2\sigma^{2}}}$$

In this equation, $G\left(x,y\right)$ is the Gaussian function, σ is the standard deviation of the Gaussian function, and $\left(x,y\right)$ represents the coordinates of the two-dimensional point within the Gaussian function.

The principle of Gaussian filtering is to scan an entire image with a window. Whenever the center of the window scans a pixel in the image, the calculation result within the neighborhood of that window is used to replace the pixel. Gaussian filtering can avoid the ringing effect and is superior to the ideal filter. If a filter has a sudden change in its frequency domain, it will cause the resulting image to become blurry at the edges.

### 3.3. Image Enhancement Algorithms

The histogram equalization algorithm adjusts the grayscale distribution of a target image based on the theory that an image is clearest when its grayscale distribution is uniform. The probability calculation formula for the grayscale histogram is shown in Equation (3).

(3) $$P\left(k\right)=\frac{n_{k}}{n},k=0,1,\ldots ,L-1$$

In this equation, $P\left(k\right)$ is the probability of the *k*-th grayscale level’s grayscale distribution, n is the total number of image pixels, and $n_{k}$ is the number of pixels in the *k*-th grayscale level.

The sum of probabilities for all grayscale levels equals 1, as shown in Equation (4).

(4) $$\sum_{k=0}^{L-1}P\left(k\right)=1$$

In this equation, $P\left(k\right)$ is the probability of the *k*-th grayscale level’s grayscale distribution and L is the total number of grayscale levels.

Histogram equalization is an algorithm that achieves the purpose of image enhancement by transforming a histogram to achieve a uniform grayscale distribution. Its basic principle is to adjust the grayscale distribution of a target image so that the grayscale distribution of the resulting image is as uniform as possible. This can improve the clarity of the recognition target, facilitating its subsequent detection by the LeNet-5 convolutional neural network.

The formula for calculating the grayscale level in the histogram equalization algorithm is as shown in Equation (5).

(5) $$S\left(k\right)=\int_{0}^{k}P\left(r\right)dr$$

In this equation, $S\left(k\right)$ is the cumulative distribution function, k stands for the first k grayscale levels in the image, and $P\left(r\right)$ represents the probability of the *r*-th grayscale level.

After applying the grayscale-level equalization algorithm, the grayscale distribution of the original image became more uniform, making the image clearer. The mapping formula for grayscale-level equalization is shown in Equation (6).

(6) $$f\left(k\right)=\left(L-1\right)*S\left(k\right)$$

In this equation, $f\left(k\right)$ is the grayscale mapping function and L is the number of grayscale levels.

The histogram equalization algorithm is fast in execution speed and has a simple implementation principle, making it suitable for deployment on embedded devices.

### 3.4. CNN Accelerator Strategy

Because a convolutional layer’s computations account for over 90% of an entire network model [37], in order to accelerate the inference process of convolutional neural networks, a strategy of loop unrolling and tiling was adopted for this paper, optimizing the convolutional layer operations within the network model. 

#### 3.4.1. Loop Unrolling

To improve the computation speed of neural networks [38], weight parameters and intermediate results were stored in Block RAM (BRAM) with high-speed read/write characteristics. Since the BRAM used for data storage only has two input/output ports and cannot access all data needed for convolutional operations at once, to overcome the limitation of memory ports and accelerate the inference process of convolutional neural networks, a strategy of loop unrolling for output feature maps was employed to optimize the convolutional layer operations in the network model.

**Figure 3:**
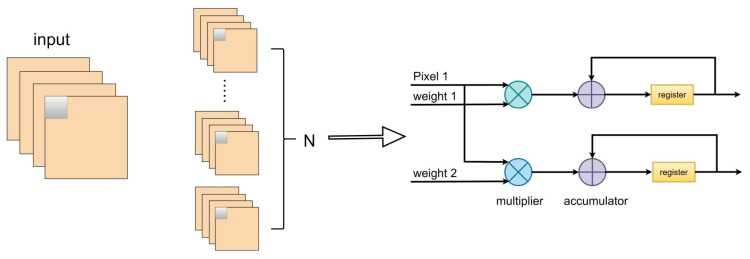
Schematic diagram of loop unrolling for output feature maps.

Loop unrolling for output feature maps was achieved by parallelizing the computation of N convolutional kernel weight parameter values and the pixel values from one input feature map, performing multiply–accumulate operations in each clock cycle, as illustrated in Figure 3 (N=2).

#### 3.4.2. Pipeline Design

The core of pipeline design lies in dividing a combinatorial logic system into multiple stages, inserting registers between each stage to temporarily store intermediate data. Each small module can execute in parallel, resulting in an increase in operating frequency and data throughput. Pipeline design is typically introduced when a circuit design has tight timing constraints and requires a high operating frequency. The pipeline design shortens the data path length within one clock cycle, increases data throughput, and improves the clock cycle. However, it also introduces data delay and significantly increases resource usage. It is a design approach that trades resources for speed.

**Figure 4:**
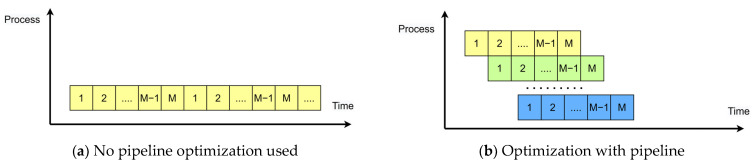
Comparison before and after adding the pipeline.

As shown in Figure 4a, it represents a basic loop with M operations and N iterations. If each operation in the loop takes one clock cycle, the total latency of the entire loop is M × N cycles. This structure uses the fewest resources but has the longest execution time. Figure 4b illustrates an ideal pipeline structure with N stages. The pipeline stages can work simultaneously, but they require a certain amount of storage resources. The throughput of this pipeline architecture D was calculated as shown in Equation (7).

(7) $$D=\frac{N}{T}=\frac{N}{\sum_{i=1}^{M+N-1}t\left(i\right)}$$

In this equation, N represents the number of iterations, M is the total number of computations, and T is the total time spent on all computations. The value $t\left(i\right)$ is the maximum processing time spent on the *i*-th computation process.

#### 3.4.3. Adder Tree

To speed up the network’s computation speed as much as possible, an adder tree was incorporated into the fully connected layer to improve data throughput. Since expanding all 256 loops would be resource-intensive on ZedBoard, the 256 loops were split into two sets of 16 loops each. Only one set of 16 loops was expanded, allowing for increased code parallelism in a resource-limited environment.

**Figure 5:**
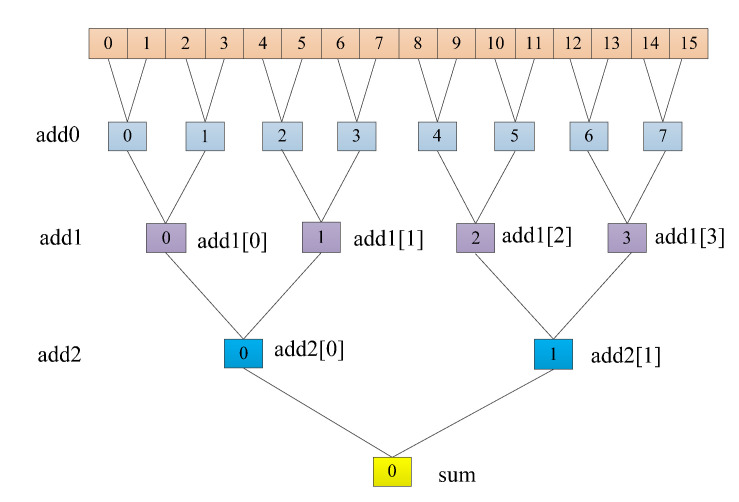
Schematic diagram of the fully connected layer’s addition tree.

In Figure 5, we can observe that 16 data points are being added. In the add0 layer, 16 data points were paired and summed in pairs using eight adders, resulting in eight values. Then, eight data points were paired and added in pairs using four adders, resulting in two values. Finally, these two values were added together using a single adder to obtain the final result sum.

## 4. Accelerator Implementation

### 4.1. Hardware Accelerator Architecture

**Figure 6:**
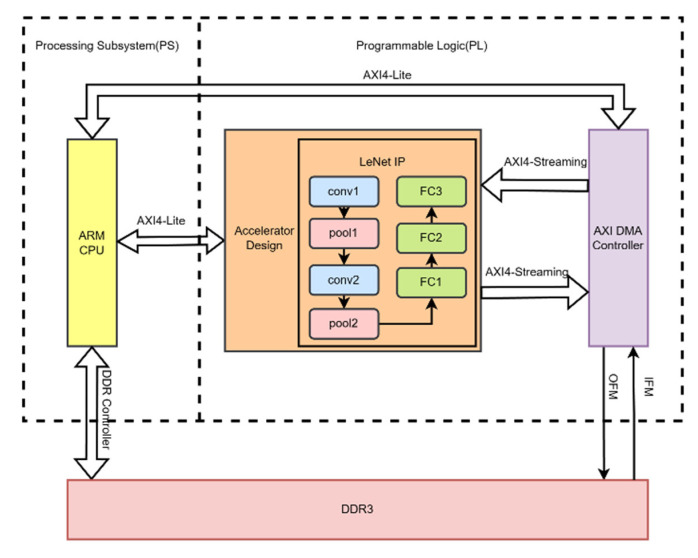
Hardware accelerator architecture diagram.

This project is based on the development of Zynq, which consists of two parts: the processing system (PS) side and the programmable logic (PL) side. The overall architecture design is shown in Figure 6. From the diagram, it can be seen that image data are stored in the DDR3 memory on the PS side, while the entire LeNet-5 convolutional neural network is implemented in the PL side. The LeNet neural network is further divided into convolution modules: pooling modules and fully connected modules. The flow of image data starts from the PS side and is transmitted to the PL side. To obtain prediction results, the PL side needs to transmit the prediction results to the PS side for display. This approach deploys the entire LeNet-5 neural network’s logic and various layers in the PL, significantly reducing data transfer and computation time, thus reducing the overall prediction time of the neural network. The resources available on the PL side of ZedBoard are sufficient to implement the LeNet neural network, which is why this method was adopted in this design to maximize network performance.

### 4.2. UNROLL Accelerator

In this approach, UNROLL statements were added to unroll the for loop. After unrolling the for loop, due to the limited input and output ports of BRAM used for storing data, it was not possible to retrieve all the data needed for convolution operations at once. To overcome this memory port limitation, ARRAY_PARTITION statements were used to partition arrays into multiple arrays based on the actual requirements. This increased the data read ports, facilitating faster data retrieval and significantly improving data throughput.

After unrolling the for loop, to address the limitation of reading only one operand in a single clock cycle and make full use of FPGA’s parallel processing capabilities, the following optimization statement was used to further partition the input array in the first convolution operation. The statement “#pragma HLS” indicates the use of optimization directives, and “variable” specifies which variable to partition. The optimization statement used was the following:

#pragma HLS ARRAY_PARTITION variable = Kw complete dim = 1

#pragma HLS ARRAY_PARTITION variable = Kw complete dim = 2

#pragma HLS ARRAY_PARTITION variable = Kb complete dim = 1

#pragma HLS ARRAY_PARTITION variable = out complete dim = 3

**Figure 7:**
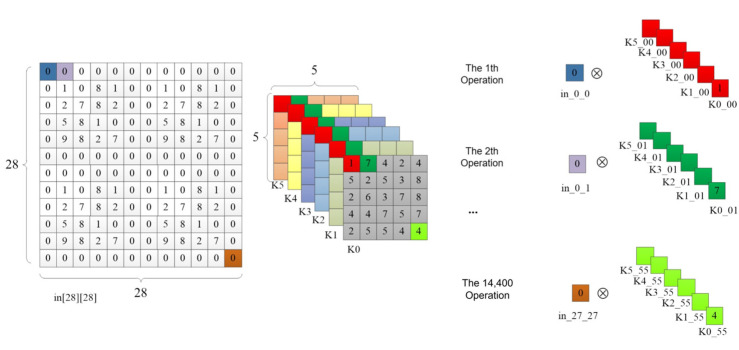
The operations after unrolling the for loop in the C1 layer.

In the 6th line of code in Table 2, the UNROLL optimization statement was used to unroll the innermost for loop of the convolution operation. The C synthesis tool generated six multipliers and performed parallel computation of these six multiplication operations, as illustrated in Figure 7. In the first operation, all six elements of the first convolution kernel were taken at once, and they were multiplied by the value of the first pixel point in_0_0 in the input feature map. In the second operation, all the first row’s second elements of the six convolution kernels were taken and multiplied by the pixel value of the second pixel in the first row of the input feature map, and so on. After 14,400 operations, all the pixel points in the input feature map had undergone multiplication operations. The output feature map size of the first convolutional layer was 24 × 24 × 6, and each convolution kernel had 25 elements, so the last multiplication operation on the input feature map’s last pixel with the last element of the convolution kernel was located at 24 × 24 × 25 = 14,400. The code optimization principle in the 12th line of Table 2 is the same, but it performed addition operations on each pixel point in the output feature map instead of multiplication. The same optimization approach was used for the convolution computation in the C3 layer.

### 4.3. PIPELINE Accelerator

A PIPELINE optimization statement instructs a compiler to pipeline code within a specified for loop, enabling multiple iterations to be executed in parallel. The II (initiation interval) parameter determines the number of clock cycles needed between iterations, i.e., the interval before the current loop can accept new data. If not explicitly set, the default value is 1, indicating fully pipelined operation. The PIPELINE optimization statement allowed us to set the II parameter to control the interval between iterations. When II = 1, it optimized the code according to the most efficient standard. If the code could not be completed within one clock cycle with II = 1, the system automatically increased II, incrementally, until the condition was satisfied. This helps balance performance and resource utilization in FPGA designs by adjusting the pipeline initiation interval as needed.

The code optimized using PIPELINE for the first convolutional layer was as shown in Table 3. Due to the “#pragma HLS PIPELINE” statement, the for loop in line 6 unrolled, and the code in line 7 was pipelined. This effect was the same for line 11. The second convolutional layer was also optimized using the same method. Similarly, to increase BRAM read/write ports and speed up data access, we needed to partition the array using the same statement.

### 4.4. Fixed-Point Parameters

In order to conserve resources on ZedBoard and reduce both the parameter count and computational complexity of CNNs, as well as the hardware resource requirements during algorithm implementation, researchers often perform quantization or fixed-point representation [39] of parameters such as weights and biases in the CNN inference process, while ensuring algorithm accuracy. The IEEE 754 standard defines the single-precision floating-point number format as shown in Equation (8).

(8) $$V={\left(-1\right)}^{s}\times M\times 2^{E-127}$$

In this equation, s is used to control the sign bit. When s is 1, V is negative, and when s is 0, V is positive. M represents the fractional part after the decimal point, and E is the exponent part, also known as the bias value. For double-precision floating-point numbers, we changed E−127 to E−1023 and left everything else unchanged. As for single-precision floating-point numbers, their data format consists of 32 binary bits. The highest bit is the sign bit, followed by an 8-bit exponent part, and the final 23 bits are the fractional part. In contrast, the decimal point in fixed-point numbers can be changed and adjusted within the program based on design requirements.

To explore the impact of different data types on FPGA resource consumption, we conducted independent experiments using fully connected layers, defining the weight parameters as floating-point numbers, integers, and fixed-point numbers. Defining integers refers to taking only the integer part of the weight parameter, ignoring the decimal part. The fixed-point numbers we used are represented using a 16-bit fixed-point notation, where 8 bits represent the integer part and the remaining bits represent the decimal part. Table 4 shows the resource consumption when weight parameters were defined as floating-point numbers, integers, and fixed-point numbers. It is evident that among these three data types, floating-point numbers consumed the most FPGA internal resources, with the number of DSPs exceeding the total resources inside ZedBoard by a large margin. Next were fixed-point numbers, which, compared to floating-point numbers, reduced BRAM consumption by 23%, DSP consumption by 29%, FF consumption by 11%, and LUT consumption by 40%. While the number of DSPs consumed also exceeded the total number of internal DSPs in the device, it was possible to adjust the number of decimal places used to represent fractions to reduce DSP resource consumption based on actual circumstances. Therefore, choosing fixed-point numbers to define weight parameters is the most suitable method, although precision may decrease slightly, but the degree of decrease is relatively small and can be negligible.

When performing fixed-point quantization on neural network parameters, the resource consumption can vary depending on the FPGA model used. Different FPGAs have varying numbers of internal resources, and using a fixed fixed-point quantization scheme for the accelerator may not be compatible with other FPGA models or may not optimize the accelerator’s performance to the fullest. To facilitate portability across different FPGA models, this design optimizes fixed-point quantization to parameterized fixed-point quantization. During the accelerator design process, the data’s fixed-point length is defined as N using macros. With this optimization, when porting the accelerator to other FPGA platforms, you only need to modify the parameter N before synthesis to maximize the accelerator’s performance based on the platform’s resource constraints.

## 5. Experimental Evaluation

This project studied the implementation of two different LeNet-5 accelerators using Xilinx’s Vivado HLS 2018.3: the UNROLL accelerator and the PIPELINE accelerator. The experimental platform used Xilinx ZedBoard, which uses the Zynq XC7Z020-CLG484-1 as the main chip and is equipped with 512 MB DDR and 220 DSP units. 

**Figure 8:**
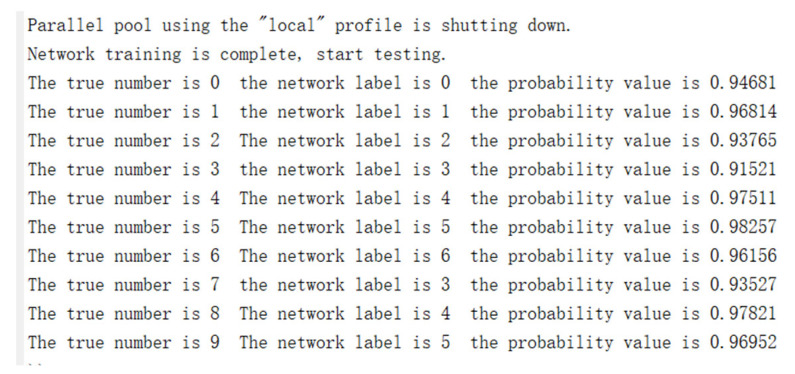
The results of MATLAB code running.

In terms of model training, this study used MATLAB as the training tool. The dataset consisted of 10 categories, with 300 training images and 10 validation images per category. The learning rate was set to 0.5 and the epoch was set to 100. The results of the MATLAB code run are shown in the Figure 8.

**Figure 9:**
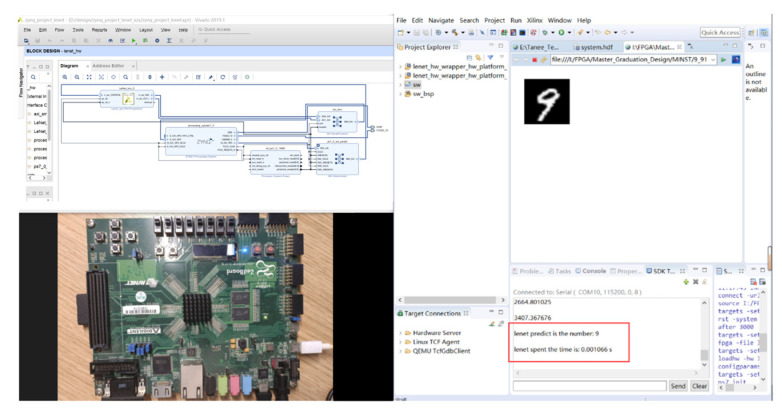
The experimental results of the accelerator.

After model training, we added the weight information to the Vivado SDK project in the form of an a header file. We implemented the inference process of the LeNet-5 network on the ZedBoard development platform, and the results are shown in Figure 9. We analyzed the effectiveness of the proposed optimization techniques through experimental data, evaluated the resource utilization (BRAM, DSP, FF, LUT) for each optimization method, and compared the performance metrics (including computation speed and power consumption) with other computing platforms.

Table 5 presents the performance and resource consumption of different approaches. It is quite evident that compared to the unoptimized accelerator, the UNROLL-optimized accelerator experienced a 25.64% increase in BRAM usage, a 1020% increase in DSP usage, a 401.99% increase in FF usage, and a 316.67% increase in LUT usage. For the PIPELINE-optimized accelerator, BRAM usage increased by 30.77%, DSP usage increased by 1670%, FF usage increased by 557.67%, and LUT usage increased by 500.44%. In terms of performance, the PIPELINE-optimized accelerator was 15.97 times faster than the UNROLL-optimized accelerator while increasing power consumption by 0.164 watts. This improvement was due to the PIPELINE-optimized accelerator’s approach of enhancing code parallelism on top of loop unrolling. It employs multiple DSP units in parallel to perform computations simultaneously, effectively breaking down a large task into many smaller subtasks allocated to DSP units for collaborative execution, which is akin to pipelining operations [40,41]. Registers were inserted between these subtasks, allowing intermediate data to be stored in these registers, thereby significantly improving data throughput.

**Figure 10:**
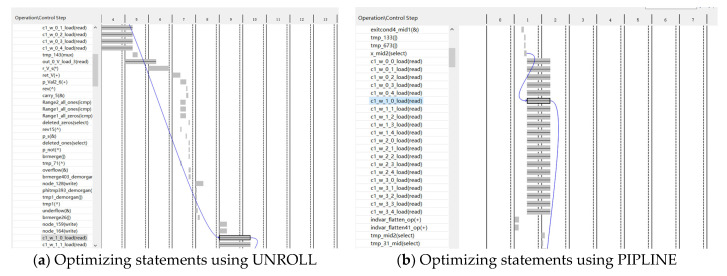
Data loading after using different optimization statements.

Figure 10 demonstrates the data processing process of the for loop after using UNROLL and PIPELINE optimization statements. Variables ending with “_load” on the left represent data read operations, while the corresponding values on the right indicate the time required for the read operation. Figure 10a illustrates the data processing process after UNROLL optimization, which revealed a time gap of more than three cycles between the read operations of data c1_w_0 and c1_w_1. Conversely, Figure 10b presents the data processing process after PIPELINE optimization, indicating that data read operations from BRAM occurred simultaneously. Hence, compared to UNROLL optimization, the PIPELINE-optimized accelerator exhibited a lower latency.

We also developed test and validation software programs on a CPU. In our related work, we compared the time and power consumption for predicting a single image in the MNIST test set (10,000 images). Table 6 shows that, under the same design functionality, the PIPELINE-optimized accelerator was 70.387 times faster than the i7-10875H CPU @2.30GHz using MATLAB computations, and it reduced power consumption by 91.37%. Table 6 also presents results from previous studies on GPUs, which indicated that the PIPELINE-optimized accelerator reduced power consumption by 93.35% compared to Nvidia GTX 840 M using a cuDNN [42], with only a slight decrease in speed (0.83 ms). This comparison demonstrated that FPGA platforms have significantly lower power consumption compared to CPUs and GPUs, resulting in substantial energy savings while maintaining excellent acceleration performance.

## 6. Conclusions

In this paper, we focused on the LeNet-5 model, investigating its structural principles and hardware implementation. We proposed a lightweight, fully programmable SOC platform design based on the ZYNQ 7000 series ZedBoard. In this work, we introduced two optimization strategies for a CNN and compared their performance. We achieved the deployment of the LeNet-5 CNN on ZedBoard through collaborative software–hardware optimization. The experimental results demonstrated that the PIPELINE-optimized accelerator had excellent performance, with a prediction time of only 1.07 ms, an error rate of 0.99%, and power consumption of 2.193 w. Compared to the i7-10875H CPU, the accelerator showed a 98.6% increase in throughput and a 91.37% reduction in power consumption. This design achieved strong performance with lower power consumption and hardware resource usage, making it highly significant for the edge deployment of CNNs with limited resources.

While this research achieved the expected results, there are some limitations to the current design that need further refinement and improvement in future work. These limitations and areas for improvement include the following:

- The separation of network training on a CPU platform and network inference acceleration on an FPGA platform can be improved for a more integrated system. Future work should focus on accelerating the backpropagation process to enhance the system’s completeness.
- Most FPGA platforms operate at frequencies ranging from 100 to 300 MHz. In this design, a frequency of 100 MHz was used to ensure correct data transfer. Optimizations can be applied to data transfer to increase clock frequencies.
- Exploring the fusion of multiple FPGAs, where multiple FPGAs collaborate, is an area that has not been extensively studied in this work. Many planning and allocation issues need to be addressed in this direction, making it a potential future research area.

## Figures and Tables

**Table 1:** The structure of the LeNet-5 neural network.

Layer Type	Input	Output	Kernel	Stride
Conv	28 × 28 × 1	24 × 24 × 6	5 × 5	1
Pool	24 × 24 × 6	12 × 12 × 6	2 × 2	2
Conv	12 × 12 × 6	8 × 8 × 16	5 × 5	1
Pool	8 × 8 × 16	4 × 4 × 16	2 × 2	2
FC	1 × 256	1 × 120	256 × 120	–
FC	1 × 120	1 × 84	120 × 84	–
FC	1 × 84	1 × 10	84 × 10	–

**Table 2:** Code for the first convolutional layer.

Row	Code
1	for(int i = 0; i < 24;i++){
2	for(int j = 0; j < 24; j++){
3	for(int y = 0; y < 5; y++){
4	for(int x = 0; x < 5; x++){
5	#pragma HLS PIPELINE
6	for(int k = 0; k < 6; k++){
7	out[i][j][k] += in[i + y][j + x] × Kw[k][y][x];
8	}}}}}
9	for(int i = 0; i < 24; i++){
10	for(int j = 0; j < 24; j++){
11	#pragma HLS PIPELINE
12	for(int k = 0; k < 6; k++){
13	out[i][j][k] += Kb[k];
14	}}}

**Table 3:** Code for the first convolutional layer.

Row	Code
1	for (int i = 0; i < 120; i++){
2	sum = 0;
3	for(int j_set = 0; j_set < 16; j_set++){
4	#pragma HLS PIPELINE
5	for(int j = 0; j < 16; j++){
6	tmp[j] = in[j + j_set × 16]*fc1_w[i][j + j_set × 16];
7	}
8	for(int k = 0; k < 8; k++){
9	add0[k] = tmp[k × 2] + tmp[k × 2 + 1];
10	}
11	for(int k = 0; k < 4; k++){
12	add1[k] = add0[k × 2] + add0[k × 2 + 1];
13	}
14	for(int k = 0; k < 2; k++){
15	add2[k] = add1[k × 2] + add1[k × 2 + 1];
16	}
17	sum += add2[0] + add2[1];
18	}
19	out[i] = sum;
20	}

**Table 4:** FPGA resource consumption.

FPGA Resource	BRAM	DSP	FF	LUT
Available quantity	1090	900	437,200	218,600
Defined as floating point	260	1282	134,701	202,357
Defined as integer	0	256	17,049	5523
Defined as fixed point	0	1024	86,264	114,800
Defined as floating point	260	1282	134,701	202,357

**Table 5:** Performance comparison of accelerators.

Design	Unoptimized	UNROLL	PIPELINE
BRAM	78	98	102
DSP	10	112	177
FF	3461	17,374	22,762
LUT	6569	27,371	39,443
Power	1.874 w	2.029 w	2.193 w
Time	20.37 ms	16.02 ms	1.07 ms

**Table 6:** Performance comparison across different platforms.

Device	Time	Power	Error Rate
PIPELINE	1.07 ms	2.193 w	0.99%
UNROLL	16.02 ms	2.029 w	0.99%
xc7z020 [43]	59.4 ms	4.2 w	–
Zynq zc706 [44]	1.607 ms	10.98 w	–
Ultra96 [45]	4.6 ms	3.55 w	–
Intel Core i7 2.30 GHz	75 ms	25.43 w	0.99%
NVidia GTX 840 M [42]	0.24 ms	33 w	1.09%

## Data Availability

Data are contained within the article.
